# HEAT SHOCK TRANSCRIPTION FACTOR B2b acts as a transcriptional repressor of *VIN3*, a gene induced by long-term cold for flowering

**DOI:** 10.1038/s41598-022-15052-6

**Published:** 2022-06-29

**Authors:** Goowon Jeong, Myeongjune Jeon, Jinwoo Shin, Ilha Lee

**Affiliations:** 1 School of Biological Sciences, Seoul National University, Seoul, 08826 Korea; 2 Research Center for Plant Plasticity, Seoul National University, Seoul, 08826 Korea; 3 Present Address: Department of Molecular Biology and Centre for Computational and Integrative Biology, Massachusetts General Hospital, and Department of Genetics, Harvard Medical School, Boston, MA 02114 USA

**Keywords:** Genetics, Molecular biology, Plant sciences, Plant development, Plant genetics, Plant molecular biology, Plant physiology

## Abstract

Vernalization, an acceleration of flowering after long-term winter cold, is an intensively studied flowering mechanism in winter annual plants. In *Arabidopsis*, Polycomb Repressive Complex 2 (PRC2)-mediated suppression of the strong floral repressor, *FLOWERING LOCUS C* (*FLC*), is critical for vernalization and a PHD finger domain protein, VERNALIZATION INSENSITIVE 3 (VIN3), recruits PRC2 on *FLC* chromatin. The level of *VIN3* was found to gradually increase in proportion to the length of cold period during vernalization. However, how plants finely regulate *VIN3* expression according to the cold environment has not been completely elucidated. As a result, we performed EMS mutagenesis using a transgenic line with a minimal promoter of *VIN3* fused to the *GUS* reporter gene, and isolated a mutant, *hyperactivation of VIN3 1* (*hov1*), which showed increased GUS signal and endogenous *VIN3* transcript levels. Using positional cloning combined with whole-genome resequencing, we found that *hov1* carries a nonsense mutation, leading to a premature stop codon on the *HEAT SHOCK TRANSCRIPTION FACTOR B2b* (*HsfB2b*), which encodes a repressive heat shock transcription factor*.* HsfB2b directly binds to the *VIN3* promoter, and *HsfB2b* overexpression leads to reduced acceleration of flowering after vernalization. Collectively, our findings reveal a novel fine-tuning mechanism to regulate *VIN3* for proper vernalization response**.**

## Introduction

As sessile organisms, plants evolve to adapt to their surrounding environment. As the transition from the vegetative to reproductive phase is usually irreversible, the proper decision of flowering time in response to the environment is one of the most important developmental processes in plants^1^. Vernalization, an acceleration of flowering after long-term winter cold, is one of the mechanisms that render plants to flower in a timely manner. *Arabidopsis* winter annuals exhibit a late-flowering phenotype but their flowering time is dramatically accelerated by vernalization. In contrast, summer annuals exhibit an early-flowering phenotype regardless of cold treatment^2^. Before winter, the winter annuals display strong expression of *FLOWERING LOCUS C* (*FLC*), a MADS-box transcription factor that represses precocious flowering, however, *FLC* is gradually suppressed according to the winter cold period, which allows plants to flower in the spring^2–4^. Thus, the molecular mechanism of vernalization in *Arabidopsis* involves the suppression of *FLC* by winter cold. Suppression of *FLC* by long-term cold exposure involves epigenetic silencing which undergoes three critical stages; stages before, during, and after cold. During stage before cold, the proteins containing plant specific B3 DNA-binding domain, VP1/ABI3-LIKE 1 (VAL1) and VP1/ABI3-LIKE 2 (VAL2) directly bind to the, so called, RY element in the 1st intron of *FLC*^5,6^*.* VAL1 and VAL2 establish the nucleation region for histone modification marks, which is a prerequisite for the next stage. In stage during cold, H3K27me3 mark, a repressive histone modification, is accumulated on the nucleation region of *FLC* chromatin by a protein complex called PHD-PRC2 complex. It includes the core components of PRC2, CURLY LEAF (CLF) and SWINGER (SWN), *Arabidopsis* homologues of H3K27 methyltransferase, VERNALIZATION 2 (VRN2), FERTILIZATION INDEPENDENT ENDOSPERM (FIE), the WD‐40 domain protein MSI1, and VERNALIZATION INSENSITIVE3 (VIN3), a protein bearing a Plant Homeo Domain (PHD) motif^7–10^. During stage after cold, accumulated H3K27me3 on the nucleation region spreads all over the gene body by LIKE HETEROCHROMATIN PROTEIN 1 (LHP1), which causes the suppression stabilized^7,11^.

Among the genes encoding the components of PHD-PRC2 complex, *VIN3* is the only gene induced by vernalization. Until exposed to cold temperature, *VIN3* is known to be expressed rarely and sparsely throughout the meristematic regions^8^. If plants are exposed to cold temperature, *VIN3* is induced within few hours, and its expression is gradually increased in proportion to the length of the cold period^8^. However, such induction is transient such that the *VIN3* level gets reverted to non-vernalized conditions if plants are returned to warm temperature^8^. The *vin3* mutant fails to respond to vernalization treatment, while constitutive expression of *VIN3* is not sufficient for vernalization response. Therefore, these results indicate that *VIN3* is a factor required, but not sufficient for vernalization^8^. In addition to the cold exposure, there are many additional factors capable of inducing *VIN3*, such as hypoxic condition and nicotinamide treatment. But the molecular mechanism behind the *VIN3* induction is still not known yet^12,13^.

There were many efforts to understand the molecular mechanism of the *VIN3* regulation over decades. For example, epigenetic regulation has been found to be a molecular basis for gradual *VIN3* expression over long-term cold exposure^12,13^. In detail, bivalent modification of active (H3Ac, H3K36me3) and repressive (H3K27me3) histone marks on the *VIN3* chromatin is revealed as a molecular mechanism of *VIN3* induction^12,13^. In addition, transcriptional regulators also have been reported recently. For example, NAC WITH TRANSMEMBRANE MOTIF 1-LIKE 8 (NTL8) has been identified as a direct regulator of *VIN3* expression through its accumulation during long-term cold exposure^14^. Moreover, two circadian clock regulators, CIRCADIAN CLOCK ASSOCIATED 1 (CCA1) and LATE ELONGATED HYPOCOTYL (LHY)*,* were identified as direct regulators of *VIN3,* which presumably render diurnal rhythms of *VIN3* expression^15^. Such findings provide supportive explanations for *VIN3* regulation under multiple thermosensory pathways which was previously constructed by mathematical modeling of *VIN3* dynamics^16,17^. However, such findings are still insufficient to understand regulatory mechanism of *VIN3*.

Cellular proteins are easily damaged when exposed to various environmental stresses. To protect cellular proteins from such cellular stresses, most eukaryotic organisms, including plants, have evolved molecular chaperones^18^. The most well-studied molecular chaperones are heat shock proteins (HSPs), which are induced by myriads of cellular stresses as well as heat shock^19^. The activation of HSPs, a general stress response in most eukaryotic organisms, is induced by a family of transcription factors known as Heat Shock Factors (Hsfs). Hsfs act as components of signal transduction that induce the expression of *HSPs* in response to a broad range of abiotic stresses^20^. By binding to the cis-elements, called Heat Shock Elements (HSEs; inverted repeat of a basal element 5**′**-nGAAn-3**′**), which are conserved in the promoters of heat stress–inducible genes of all eukaryotes, Hsfs directly regulate the transcription of stress-responsive genes, including HSPs^21–23^. There are 21 *Hsf* genes in the *Arabidopsis* genome and are divided into three classes; A, B and C^24,25^. Class A contains the motif (AHA motif) with activation activity, which is characterized by aromatic, large hydrophobic, and acidic amino acid residues^25^. Class A proteins have been reported to act as positive regulators in response to a broad range of stress conditions in plants^22,26,27^. In contrast, class B and C proteins are considered transcriptional repressors, as they lack AHA motifs and contain the repressive R/KLFGV motif^28^. Among the five class B proteins, HsfB1 and HsfB2b have been reported to act as transcriptional repressors, but positively regulate redundantly the acquired thermotolerance, an enhanced thermotolerance by prior heat treatment^29^. Besides the acquired thermotolerance, HsfB1 and HsfB2b have been shown to negatively regulate pathogen resistance redundantly^30^, while HsfB2b alone has been shown to mediate abiotic stress responses of the circadian clock^31^.

In the present study, we identified *HsfB2b* as a novel repressor of *VIN3*. Further, we isolated one mutant, *hov1,* with hyperactive *VIN3* from a mutant pool that originated from the *pVIN3::GUS* reporter lines. The mutant was identified to carry a nonsense mutation in exon 1 of *HsfB2b*. Overexpression of *HsfB2b* rescued hyperactive *VIN3* in *hov1,* and HsfB2b was found to bind to the conserved HSEs located in the 5**′**-UTR of *VIN3*. Moreover, overexpression of *HsfB2b* in the late-flowering *FRI* Col background resulted in defects in the vernalization response, suggesting that *HsfB2b* negatively regulates the vernalization response.

## Results

### Isolation and characterization of the mutant showing hyperactivation of *VIN3*, *hov1*

**Figure 1:**
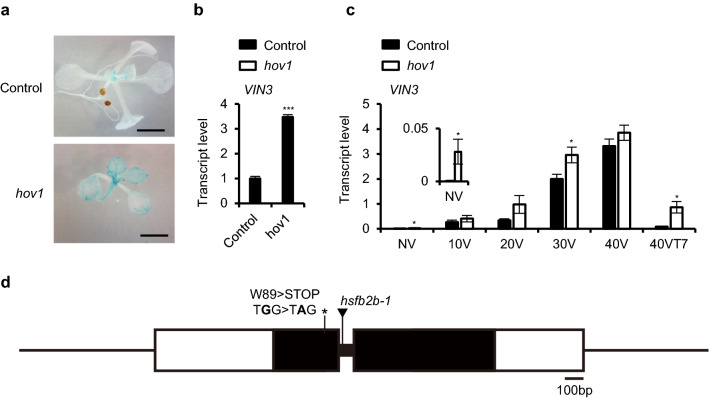
Isolation and characterization of the mutant, *hov1*, showing hyperactivation of *VIN3*. (**a**,**b**) Characterization of the *hov1* mutant. Seedlings of the control (− 0.2* kb pVIN3_U_I::GUS*) and *hov1* were grown at room temperature for 10 days and analyzed after 3 days of cold treatment. (**a**) Images of representative seedlings after GUS staining. (Scale bars, 2 mm) (**b**) Endogenous *VIN3* transcript levels in control and *hov1*. Data are presented as mean ± SEM of three biological replicates. Asterisks indicate significant difference compared with the control (Student’s t-test; ***P < 0.001). (**c**) Time-course analysis of *VIN3* levels during vernalization treatment. NV, non-vernalized; 10V, 20V, 30V, and 40V, 10 d, 20 d, 30 d, and 40 d vernalized, respectively; 40VT7, 7 d grown at room temperature after 40V. Data are presented as mean ± SEM of three biological replicates. Asterisks indicate significant difference compared with the control (Student’s t-test; *P < 0.05). The inset in (**c**) is enlarged for NV. (**d**) Schematic structure of the *HsfB2b* gene*.* Black bars indicate exons, and white boxes and lines represent untranslated regions and introns, respectively. The mutations that occurred in the two alleles are shown: T-DNA insertion as a triangle and point mutation as an asterisk.

To identify upstream regulators of *VIN3*, the GUS reporter line (− 0.2 kb *pVIN3_U_I::GUS*)^15^ was mutagenized with ethyl methanesulfonate (EMS). A total of 3,412 M1 lines were generated and their seeds were harvested as M2 seeds. Approximately 25 M2 seedlings from each line were grown at room temperature for 10 days, and transferred to the cold chamber for 3 days, then analyzed for GUS staining. Throughout the screening, the first and second true leaves of the M2 seedlings were used for GUS staining when seedlings produced more than 5 leaves. One mutant showing the hyperactivation of GUS was identified and initially named *hov1*. Compared to the parental line, which showed a very weak GUS signal after 3 days of cold exposure (3V), *hov1* showed an enhanced GUS signal with the same treatment (Fig. 1a). Consistent with the results of the GUS assay, endogenous *VIN3* transcript levels were enhanced in the mutant after 3V (Fig. 1b). The mutant phenotype was not found in the F1 plants when backcrossed to the parental line, and the phenotype was segregated by approximately 3:1 (81 WT vs 29 mutants) in F2 population. Such results indicate that the mutant phenotype is completely recessive and is caused by a mutation in a single locus. Thereafter, we performed a time course analysis of *VIN3* levels in *hov1* for vernalization treatment*.* As shown in Fig. 1c, *hov1* plants displayed higher levels of *VIN3* than the controls without vernalization treatment, indicating that *HOV1* is necessary to completely suppress *VIN3* at room temperature. The *hov1* plants also had higher levels of *VIN3* throughout the vernalization time course and the mutant had higher levels of *VIN3* than the control plants after returning to room temperature for 5 days (40VT5). These results indicate that *HOV1* is required for the suppression of *VIN3* under all conditions.

To identify the causative mutation, *hov1* was crossed with L*er* for positional cloning. A total of 156 F2 plants with enhanced GUS signals in the leaves were selected for mapping analysis. We mapped the mutation to the 590 kilobase pair interval on chromosome 4, which contained 142 genes (Supplementary Fig. S1). The genomes of *hov1* and parental  − 0.2* kb pVIN3_U_I::GUS* were sequenced using the Illumina sequencing method for comparison. Analysis of the sequence data revealed nine potentially disruptive point mutations, including one mutation within the At4g11660 gene (G to A, causing a nonsense mutation from Trp89 to the stop codon) (Fig. 1d). At4g11660 encodes the class B heat shock transcription factor, HEAT SHOCK TRANSCRIPTION FACTOR B2b (HsfB2b).

### *HsfB2b* acts as a transcriptional repressor of *VIN3*

**Figure 2:**
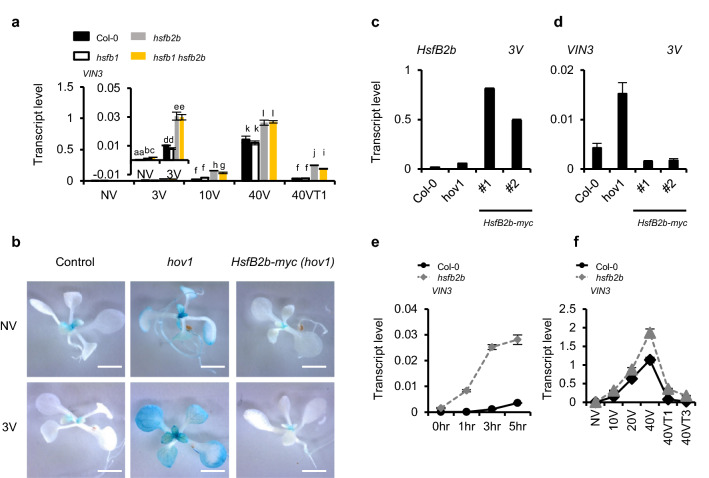
HsfB2b acts as a transcriptional repressor of *VIN3*. (**a**) *HsfB1* is not functionally redundant with *HsfB2b* in *VIN3* regulation. *VIN3* transcript levels in Col-0, *hsfb1, hsfb2b* and *hsfb1 hsfb2b* during vernalization treatment were determined using RT-qPCR. NV, non-vernalized; 3V, 10V, 40V, 3, 10, and 40 d vernalized; 40VT1, 1 d growth at room temperature after 40V. Transcript levels were normalized to those of *PP2A*. Data are presented as mean ± SEM of three biological replicates. Significant differences have been marked using different letters (a-l; *P* < 0.05; one-way ANOVA followed by Tukey’s post-hoc test). The inset in (**a**) is enlarged for the NV and 3V. (**b**,**d**) Complementation of *hov1* with *pHsfB2b::HsfB2b-myc*. (**b**) GUS staining of NV or 3V seedlings of the parental line, *hov1*, and *pHsfB2b::HsfB2b-myc hov1* transgenic line. Images of representative seedlings after GUS staining (**c**) *HsfB2b* or (**d**) *VIN3* transcript levels in 3V Col-0, *hov1*, and two representative transgenic lines of *pHsfB2b::HsfB2b-myc hov1*. Transcript levels were normalized to those of *PP2A*. Data are presented as mean ± SD of three technical replicates. (**e**,**f**) Effects of *hsfb2b* mutation on *VIN3* levels. *VIN3* levels in Col-0 and *hsfb2b*, as determined by RT-qPCR after (**e**) short-term cold treatment (0, 1, 3, and 5 h) or (**f**) long-term cold exposure (NV, 10V, 20V, 40V, 40VT1, 40VT3). Transcript levels were normalized to those of *PP2A*. Data are presented as mean ± SD of three technical replicates.

To verify that *HsfB2b* is the causative gene of the upregulation of *VIN3* level in *hov1*, we checked the *VIN3* level in the T-DNA-inserted mutant, *hsfb2b-1.* As expected, *hsfb2b-1* had higher levels of *VIN3* than wild-type Col-0 under non-vernalized conditions (Fig. 2a). Previously, *HsfB2b* was reported to display functional redundancy with *HsfB1* instead of *HsfB2a* for acquired thermotolerance, although the sequence of *HsfB2b* had higher homology with that of *HsfB2a* than *HsfB1*^29^*.* To determine whether *HsfB2b* is functionally redundant with *HsfB1* for the vernalization response, we compared *VIN3* levels among the *hsfb2b, hsfb1,* and *hsfb1 hsfb2b* mutants. The *hsfb1* mutant displayed similar levels of *VIN3* to the wild-type under all conditions. However, the *hsfb1 hsfb2b* double mutant did not show any difference compared with *hsfb2b* in *VIN3* levels (Fig. 2a)*.* Such finding suggests that *HsfB1* is not functionally redundant to *HsfB2b*, at least for *VIN3* regulation.

Finally, we introduced *HsfB2b::HsfB2b-myc* into the *hov1* mutant to determine whether *HsfB2b* can rescue the *hov1* mutation. The transcript levels of *HsfB2b* were found to be overexpressed in all transgenic lines we obtained (Figs. 2c, 6a, and d). Here, we used two representative lines of *HsfB2b:HsfB2b-myc hov1,* #1, and #2. As expected, the phenotype of the GUS signal in *hov1* was complemented by *HsfB2b::HsfB2b-myc,* such that the GUS signal was barely detected after 3 d of cold exposure (Fig. 2b). Moreover, the endogenous *VIN3* transcript levels in both transgenic lines were lower than those in Col-0, as well as the *hov1* mutant, whereas *HsfB2b* transcript levels in the transgenic lines were higher than those in Col-0 (Fig. 2c and d)*.* Taken together, our results indicate that *HsfB2b* is a causative gene that reduces *VIN3* level in *hov1* and acts as a transcriptional repressor of *VIN3.*

In a previous report, *VIN3* was found to gradually increase by long-term cold exposure from the first day of cold treatment^13^. Thus, we determined whether HsfB2b affects *VIN3* expression during the initial stage of vernalization treatment (Fig. 2e and f). As shown, *hsfb2b* caused strong derepression of *VIN3* from the initial phase, and the effect was strongest after 3 h of cold treatment. Of note, the derepression effect of *hsfb2b* is stronger at short-term cold and 40VT1 (1 d at room temperature after returning from 40 days of vernalization treatment) than during vernalization treatment (Fig. 2e and f).

### Effects of vernalization on HsfB2b

**Figure 3:**
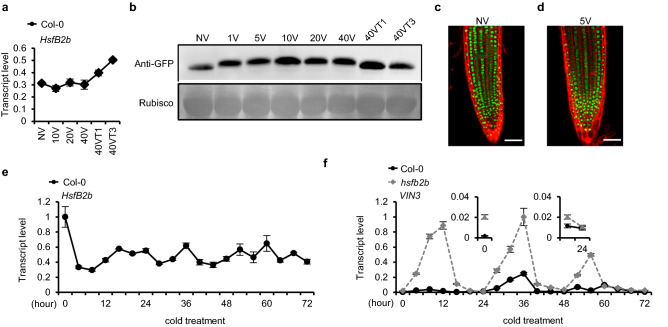
Characterization of *Hsfb2b* during cold and vernalization treatments. (**a**) Effect of vernalization treatment on the transcript levels of *HsfB2b*. Col-0 seedlings were vernalized before total RNA extraction for RT-PCR analysis. Expression levels were normalized to those of *PP2A*. Data are presented as mean ± SD of three technical replicates. (**b**) Immunoblot analysis of the HsfB2b-eGFP protein extracted from vernalized seedlings of *pHsfB2b::HsfB2b-eGFP*. Rubisco was used as a loading control. NV, non-vernalized; 1V, 5V, 10V, 20V and 40V, 1 d, 5 d, 10 d, 20 d, and 40 d, respectively; 40VT1, 40VT3, 1 d, and 3 d, grown at room temperature after 40V, respectively. Original blots are presented in Supplementary Figure S7. (**c**,**d**) Confocal images of roots from NV or 5V plants expressing *pHsfB2b::HsfB2b-eGFP*. (Scale bars, 20 μm) Five days-old *Arabidopsis* seedlings, with or without 5 days of cold exposure, were harvested and counterstained with propidium iodide. (**e**) Effect of the early phase of vernalization on the rhythmic expression of *HsfB2b* in Col-0*.* Expression levels were normalized to those of *PP2A*. Data are presented as mean ± SD of three technical replicates. (**f**) Effect of the *hsfb2b* mutation on the rhythmic expression of *VIN3*. *VIN3* levels during the early phase of vernalization were analyzed using seedlings of Col-0 and *hsfb2b* collected at 4-h intervals over 72 h in LD at 4 °C. The x-axis indicates the exposure time to cold. Data are presented as mean ± SD of three technical replicates.

*Hsfb2b* expression is well-known to be induced by heat shock treatment to suppress hyperactivated heat shock-responsive genes^29^. However, the effect of long-term cold treatment on *Hsfb2b* is unknown. Before vernalization treatment, the basal level of *Hsfb2b* was detected as previously reported (Fig. 3a). During the long-term cold treatment, such levels of *Hsfb2b* did not change significantly. In contrast, a slight increase of *Hsfb2b* was observed when returned to room temperature (Fig. 3a). We also checked HsfB2b protein levels using *pHsfB2b::HsfB2b-eGFP* transgenic lines during vernalization treatment (Fig. 3b). However, the protein levels were not found to be significantly affected by vernalization treatment. Nonetheless, vernalization treatment caused retarded migration of HsfB2b-eGFP proteins on polyacrylamide gel from the initial phase, suggesting that HsfB2b undergoes post-translational modifications, such as phosphorylation by cold (Fig. 3b). Such retardation in HsfB2b migration was also observed in the protein immunoblot result using *pHsfB2b::HsfB2b-myc* transgenic plants (Supplementary Fig. S2). After returning to room temperature, such modifications may have been rapidly erased as 40VT1 displayed the same protein pattern as NV.

To confirm whether the retarded migration of HsfB2b protein is due to the phosphorylation, phosphatase assay was conducted using total protein extracted from vernalized or non-vernalized *pHsfB2b::HsfB2b-myc* seedlings. Subsequent immunoblot assay showed that the retarded migration of HsfB2b-myc from vernalized seedlings was abolished by phosphatase treatment, while migration of HsfB2b-myc from non-vernalized seedling was not changed by the treatment (Supplementary Fig. S3). These results indicate that retarded migration of HsfB2b-myc from vernalized seedlings was due to the phosphorylation.

As the cellular localization of other Hsf is changed by post-translational modification^32^, we checked whether cold treatment can change that of HsfB2b (Fig. 3c and d). Using the *pHsfB2b::HsfB2b-eGFP* transgenic lines, we observed the root tissue before and after 5 days of cold. In both cases, GFP signals were observed in the nucleus, indicating that neither cold treatment nor protein modification altered the cellular localization of HsfB2b. This result is consistent with the fact that the protein sequence of HsfB2b has a nuclear localization signal (NLS), but lacks a nuclear export signal (NES) motif^25^. Taken together, *HsfB2b* is neither transcriptionally induced nor is the subcellular localization of the proteins altered by vernalization treatment.

*VIN3* expression is reported to show a circadian rhythm and *HsfB2b* acts as a negative regulator of the circadian clock regulator, *PSEUDO RESPONSE REGULATOR7* (*PRR7)*^15,16,31^. Thus, we verified whether the *VIN3* rhythm was affected by *hsfb2b* during cold treatment. Although the amplitude of the circadian rhythm was increased by *hsfb2b* mutation due to the increase in *VIN3* level, the rhythmic pattern was not significantly different (Fig. 3e and f). Therefore, *HsfB2b* seems to constitutively repress *VIN3,* and this repression is independent of the *HsfB2b*-regulated circadian clock.

### HsfB2b directly regulates *VIN3* repression

**Figure 4:**
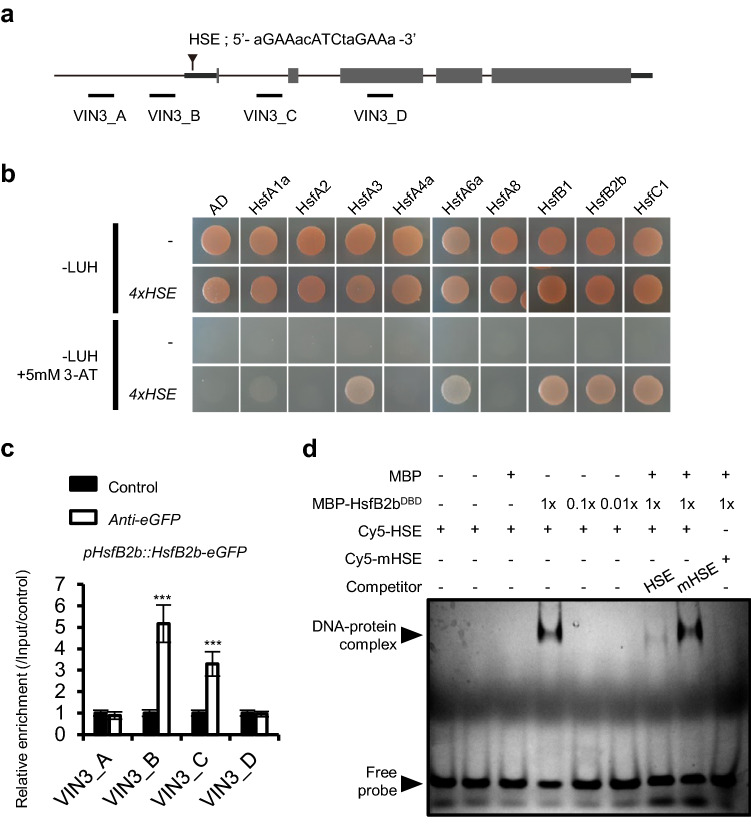
HsfB2b directly binds to the HSE on the *VIN3* gene. (**a**) Schematic of the *VIN3* gene with description of the HSE_VIN3_ sequence and PCR amplicons. A-D, amplicons used for ChIP-qPCR. Black bars indicate untranslated region, and grey boxes and lines represent exons and introns, respectively. (**b**) Yeast one-hybrid assay between heat shock factors and HSE_VIN3_ cis-element. As DNA baits, four tandem copies of the 24-bp sequences containing HSE_VIN3_ were inserted into the *pHisI* vector and used as the reporter construct. The CDS of *HsfA1a*, *HsfA2*, *HsfA3*, *HsfA4a*, *HsfA6a*, *HsfA8*, *HsfB1*, *HsfB2b*, and *HsfC1* was cloned into *pGADT7* and used as an effector construct. GAL4 AD alone (AD) was used as the control. The effector and reporter constructs were co-transformed into the yeast strain, AH109. Representative growth status of yeast cells is shown on synthetic defined (SD)-LUH medium, with or without 5 mM 3-AT. LUH, SD medium without Leu, Ura, His; − LUH + 5 mM 3-AT, SD medium without Leu, Ura, His but containing 5 mM 3-Amino-1,2,4-triazole. (**c**) ChIP-qPCR showing the enrichment of HsfB2b-eGFP. Chromatin of the transgenic line expressing *pHsfB2b::HsfB2b-eGFP* was immunoprecipitated using control beads or GFP-trap beads. Histograms show mean values ± SEM (*n* = 2 biological replicates, each biological replicate is an average value of three technical replicates) for enrichment calculated by percent input normalized against the control. Asterisks indicate significant differences compared with the control (Student’s t-test; ***P < 0.001). (**d**) In vitro binding of the recombinant MBP-HsfB2b^DBD^ to HSE_VIN3_ sequence by EMSA. Purified recombinant MBP-HsfB2b^DBD^ or MBP was incubated with Cy5-labeled 40-bp sequences including *HSE*_*VIN3*_ element as HSE probes. The same sequences with mutations in the *HSE*_*VIN3*_ was used as control probe (mHSE). Unlabeled competitor DNA (100 × molar excess) was added to each reaction, as indicated. Original gels are presented in Supplementary Figure S8.

Heat shock transcription factors regulate a variety of genes by directly binding to the HSE in the promoters^22,23^. Consistently, HSE was detected near the *VIN3* promoter, approximately 40-bp downstream of the transcription start site (Fig. 4a). In addition, the HSE was highly conserved among the *VIN3* orthologs from Brassicaceae species (*Arabidopsis thaliana*, *Arabidopsis lyrata, Boechera stricta*, and *Capsella rubella*) (Supplementary Fig. S4). Therefore, we determined whether HsfB2b directly binds to the *VIN3* promoter. In silico analyses using the DNA affinity purification (DAP)-seq database^33^ showed that several heat shock transcription factors bind to the 5**′**-UTR of *VIN3*, where HSE is located (Supplementary Fig. S5).

We proceeded to assess whether HsfB2b bound to HSE_VIN3_ using yeast one-hybrid assay (Fig. 4b). Among the nine Hsf proteins analyzed, HsfA1, HsfA6a, HsfB1, HsfB2b, and HsfC1 were found to interact with HSE_VIN3_. To confirm *in planta* binding, we also performed chromatin immunoprecipitation-qPCR using transgenic *pHsfB2B::HsfB2b-eGFP,* grown under long days without cold treatment (Fig. 4a and c). HsfB2b-eGFP proteins were found to be enriched in the promoter region near the HSE location even without cold treatment, which is consistent with the elevated *VIN3* level in *hsfb2b*. We also conducted electrophoretic mobility-shift assay (EMSA) to assess whether HsfB2b specifically binds to HSE_VIN3_ DNA element in vitro. The purified recombinant protein, MBP-HsfB2b^DBD^, DNA binding domain of HsfB2b fused with maltose binding protein (MBP), from *E. coli* indeed binds the HSE_VIN3_ probe but fails to bind the mutated version of HSE_VIN3_ probe (Fig. 4d). Taken together, these data strongly support the hypothesis that HsfB2b directly regulates *VIN3* repression.

### *hsfb2b* mutation does not change vernalization response under normal condition

**Figure 5:**
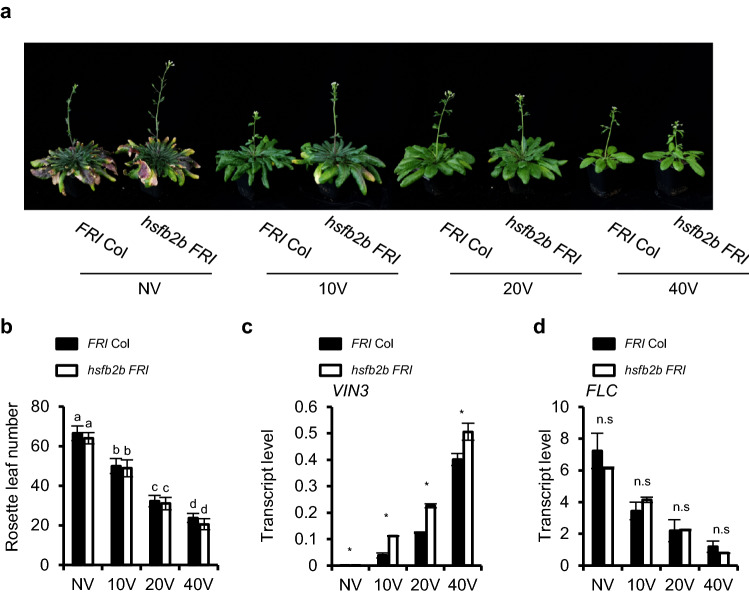
*hsfb2b* mutation does not change vernalization response under normal condition. (**a**) Images of *FRI* Col and *hsfb2b FRI* without (NV) or with 10 d (10V), 20 d (20V), 40 d (40V) of vernalization. (**b**) Flowering time of *FRI* Col and *hsfb2b FRI* after vernalization*.* Flowering time was measured by counting the number of primary rosette leaves formed when the first flower opened. Data are presented as mean ± SD. Significant differences have been marked using different letters (a-l; P < 0.05; one-way ANOVA followed by Tukey’s post-hoc test). (**c**,**d**) *VIN3* or *FLC* transcript levels in *FRI* Col and *hsfb2b FRI* after vernalization treatment. The transcript levels were normalized to those of *PP2A*. Data are presented as mean ± SD of three technical replicates. Asterisks indicate significant difference compared with the control (Student’s t-test; *P < 0.05). n.s, not significant.

To analyze the effect of *hsfb2b* on vernalization response, the *hsfb2b* mutation was introduced into *FRI* Col, a vernalization-sensitive line, by genetic cross^34^. As shown in Fig. 5, the flowering time of *hsfb2b FRI* was similar to that *of FRI* Col, although *VIN3* levels were higher in *hsfb2b FRI* than in *FRI* Col throughout the time course of vernalization treatment (Fig. 5a–c). Consistently, the *FLC* levels were not significantly different between the two genotypes throughout vernalization treatment (Fig. 5d). Thus, the increased levels of *VIN3* in *hsfb2b* may not alter the vernalization response under normal growth conditions. Such findings suggest that *VIN3* levels in the *FRI* Col are sufficient for a proper vernalization response.

### *HsfB2b* overexpression leads to hyposensitive response to vernalization

**Figure 6:**
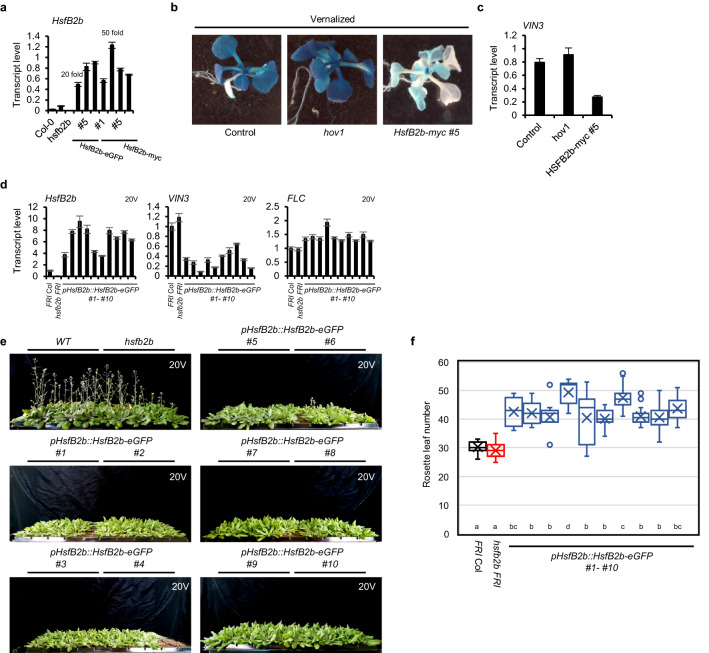
*HsfB2b* overexpression leads to hyposensitive response to vernalization. (**a**) Comparison of the *HsfB2b* transcript levels in Col-0, *hov1*, *hsfb2b,* and the transgenic lines expressing *pHsfB2b::HsfB2b-eGFP* or *pHsfB2b::HsfB2b-myc* in *hov1*. Transcript levels were normalized to those of *PP2A*. Fold changes relative to Col-0 were marked for comparison. Data are presented as mean ± SD of three technical replicates. (**b**) GUS staining for 40V seedlings of the parental lines (*− *0.2* kb pVIN3_U_I::GUS*), *hov1*, and *pHsfB2b::HsfB2b-myc hov1.* Images of representative seedlings after GUS staining are shown. (**c**) *VIN3* levels in 40V seedlings of the parental line, *hov1*, and *pHsfB2b::HsfB2b-myc hov1.* Transcript levels were normalized to those of *PP2A*. Data are presented as mean ± SD of three technical replicates. (**d**) Transcript levels of *HsfB2b*, *VIN3*, and *FLC* in *FRI* Col, *hsfb2b FRI*, and transgenics expressing *pHsfB2b::HsfB2b-eGFP* in *FRI* Col after 20V. Transcript levels were normalized to those of *UBC*. Data are presented as mean ± SD of three technical replicates. (**e**) Photographs of WT (*FRI* Col), *hsfb2b FRI*, and transgenics expressing *pHsfB2b::HsfB2b-eGFP* in *FRI* Col after 20V. Images captured when 20V WT and *hsfb2b* plants were fully flowered. (**f**) Flowering time is presented as a box plot. Flowering time was measured by counting the number of primary rosette leaves formed when the first flower opened. The center lines indicate the medians. Box limits indicate the 25th and 75th percentiles. Significant differences have been marked using different letters (a–d; *P* < 0.05; one-way ANOVA followed by Tukey’s post-hoc test).

In our complementation analysis, all *HsfB2b* transgenic lines displayed overexpression of *HsfB2b*, although the transgenes were driven by the endogenous promoter. Thus, we analyzed the vernalization response in *HsfB2b* overexpressing lines. The *hov1* mutant, containing a nonsense mutation in the first exon of *HsfB2b*, showed approximately threefold higher *HsfB2b* levels than Col-0, which might be due to the negative feedback regulation (Fig. 6a). When the transgenes, *pHsfB2b::HsfB2b-myc* or *pHsfB2b::HsfB2b-eGFP*, were introduced into the *hov1* background, the *HsfB2b* levels were increased by 20–50-fold relative to that of Col-0, indicating that the transgenic lines were *HsfB2b* overexpressors (Fig. 6a). The GUS and endogenous *VIN3* levels among the parental lines (− 0.2 *kb pVIN3_U_I::GUS*), *hov1* (in − 0.2 *kb pVIN3_U_I::GUS* background), and *pHsfB2b::HsfB2b-myc hov1* (Fig. 6b and c) were subsequently compared after 40 days of vernalization treatment. The overexpression of *HsfB2b* was found to markedly reduce *VIN3* levels after 40 days of vernalization treatment. Such finding is consistent with the hypothesis that HsfB2b represses *VIN3* transcription.

We proceeded to verify whether *HsfB2b* overexpression caused any changes in the vernalization response. Briefly, we introduced *pHsfB2b::HsfB2b-eGFP* into *FRI* Col by transformation. As expected, all 10 transgenic lines showed overexpression of *HsfB2b* (3 ~ 10 folds) based on the level after 20 days of vernalization (20V) (Fig. 6d). In these transgenic lines, *VIN3* levels were lower than those in both *FRI* Col and *hsfb2b FRI* after 20V, which supports the hypothesis that *HsfB2b* overexpression causes the repression of *VIN3* in *FRI* Col plants (Fig. 6d). Consistent with the fact that *VIN3* is required for the suppression of *FLC*^8^, the *FLC* levels in *pHsfB2b::HsfB2b-eGFP FRI* lines were slightly higher than those in *FRI* Col after 20V (Fig. 6d). Finally, the flowering time of the transgenic lines, *pHsfB2b::HsfB2b-eGFP FRI,* was less accelerated than that of both *FRI* Col and *hsfb2b FRI* by 20V (Fig. 6e and f). Therefore, in contrast to *hsfb2b* mutation, *HsfB2b* overexpression causes defects in the vernalization response under normal growth conditions.

## Discussion

*VIN3* is required for proper vernalization in *Arabidopsis*, particularly winter annuals. However, the molecular mechanism by which *VIN3* is finely regulated has not been fully elucidated. In this study, we isolated a mutant, *hov1,* that showed hyperactivation of *VIN3*. By map-based cloning combined with whole-genome resequencing, *HsfB2b* was defined as the causative gene for *VIN3* derepression in *hov1.* Interestingly, *hsfb2b* exhibited higher *VIN3* levels under all conditions, including before and after vernalization. Therefore, *HsfB2b* might act as a general repressor of *VIN3*, regardless of cold treatment. Nonetheless, the intensity of the derepression in *hsfb2b* was strongest at the initial stages of cold treatment and stronger at the phase of return to room temperature after 40V than during vernalization treatment. Taken together, *HsfB2b* might act in a fine-tuning mechanism, suppressing precocious *VIN3* activation during the fall when temperature drops abruptly and suppressing *VIN3* levels rapidly after spring.

Higher *VIN3* levels in *hsfb2b* failed to show a stronger vernalization response in the late-flowering *FRI* Col background. This result is consistent with that of previous studies where ectopic expression of *VIN3* was not found to alter the vernalization response, despite complementing *vin3* mutation^35,36^. In contrast, the lower *VIN3* levels in the *HsfB2b* overexpression lines caused a weak vernalization response in both the acceleration of flowering time and *FLC* suppression by 20 days of vernalization treatment. This result is also consistent with the fact that the *vin3* mutation causes failure of the vernalization response^7,8^. Finally, HsfB2b was found to directly repress *VIN3,* a key factor in the vernalization process, by binding to the HSE on the 5**′**-UTR of *VIN3* (Fig. 5). Of note, the HSE_VIN3_ sequences on the 5**′**-UTR of the *VIN3* orthologues are highly conserved*,* whereas other regions of the 5**′**-UTR are relatively diversified among Brassicaceae species. As vernalization responses have been observed throughout Brassicaceae, conservation of such cis-elements suggests that *VIN3* regulation by Hsfs may also be conserved across the Brassicaceae family^37–39^.

Although *hsfb2b* shows constitutively up-regulated *VIN3* level, vernalization response is not much affected by the *hsfb2b* mutation. Similarly, vernalization response is not affected by the ectopic expression of *VIN3*, despite complementing *vin3* mutation^35,36^*.* Previous report indicated that vernalization-mediated removal of H3K4me3 is a prerequisite for VIN3-PRC2 accumulation at the *FLC* nucleation region. In addition, the association of VAL1 and VAL2, two B3 domain transcription factors, to the nucleation region of *FLC* is also reported as a prerequisite for VIN3-containing PRC2 activity^5,6^. Such results explain why VIN3 alone is not sufficient to suppress *FLC.* In natural conditions, where daily temperature fluctuates largely, especially at late fall or early spring, it is beneficial to fine-tune vernalization response. In case the prerequisites mentioned above are fulfilled accidentally by sudden temperature drop, the fine-tuning of *VIN3* expression by transcription repressor like HsfB2b may provide another layer of huddle to prevent hypersensitive vernalization responses.

Early works on vernalization have reported that the immediate treatment of heat above 30 °C after long-term cold can erase vernalization effect. It is called devernalization^40,41^. Since overexpression of *HsfB2b* causes reduced *VIN3* expression, resulting in weak vernalization response, (Fig. 6) and *VIN3* level in 40VT1 plant is elevated in *hsfb2b* compared to wild-type (Fig. 2f), it is possible that HsfB2b may mediate devernalization process. However, we could not observe any difference in devernalization effect between wild-type and *hsfb2b* mutant when heat (30 °C) treated right after long-term cold treatment (Supplementary Fig. S6). Therefore, it is not likely that HsfB2b is involved in erasing repressive epigenetic marks, such as H3K27me3, induced by vernalization on the *FLC* locus, which is a proposed molecular mechanism of devernalization^42^.

In *Arabidopsis,* Hsfs have been reported to regulate diverse stress responses, including responses to both biotic and abiotic stresses, such as bacterial infection, fungal infection, and heat and drought stresses^43–46^. During such responses, both class A and class B Hsfs are incorporated into complex and multi-layered regulatory systems, and different combinations of Hsfs seem to act on each stress response^27^. Although most of *Hsfs* are induced by heat stress, they usually show basal expression level without heat or cold stress similar to *Hsfb2b*. Such basal expression level may be required for the rapid response to diverse stresses. Here, *HsfB1* was not functionally redundant with *HsfB2b* for *VIN3* regulation (Fig. 2). However, several Hsfs, besides HsfB2b, including HsfB1, bound to the HSE_VIN3_ elements present in the 5**′**-UTR of *VIN3* in the yeast one-hybrid assay (Fig. 5). Therefore, other Hsfs, recognizing HSE_VIN3,_ may regulate *VIN3* transcription in response to other stresses, such as low oxygen conditions at which *VIN3* is induced^47^. This notion is consistent with the finding that Hsfs are required for a broad range of stress responses^22,26,27^. It would be interesting to determine whether VIN3 acts as a hub for the stress responses mediated by Hsfs.

Plants perceive winter cold as a signal for vernalization, but simultaneously perceive it as long-term cold stress. In *Arabidopsis*, several HSPs and factors are strongly induced by cold stress, and the roles of both HSPs and Hsfs in the cellular response to cold stress have been reported previously^22,48–50^. The HsfB2b protein displayed retarded migration on polyacrylamide gels during vernalization treatment. Such cold-induced post-translational modifications indicate that *HsfB2b* is involved in a subset of cold signal transduction (Fig. 3b). Previously, HsfB2b has been reported as a protein phosphorylated by SnRK2 kinases, which is activated by plant hormone abscisic acid (ABA)^51^. Considering that ABA is known to play a role in broad range of stress response including cold response^52,53^, the phosphorylation of HsfB2b is probably involved in stress responses triggered by ABA. Moreover, the transcript level of *HsfB2b* was slightly elevated after returning from cold temperatures to warm temperatures (Fig. 3a). Such observations may indicate that *HsfB2b* is required for sensing temperature changes, which are inevitable during vernalization treatment. Thus, *VIN3* regulation by *HsfB2b* may have evolved from a mechanism that senses cold stress.

The circadian clock was previously demonstrated to be involved in the regulation of *VIN3*, and components of the circadian clock, *CCA1* and *LHY*, directly regulate the diurnal rhythm of *VIN3* during vernalization treatment^15,16^. One of the circadian clock regulators, *PRR7*, has also been reported to be a transcription factor repressed by *HsfB2b*^31^*,* which is required for proper abiotic stress responses. However, our data indicate that *HsfB2b* is not involved in regulating the diurnal rhythm of *VIN3* under cold treatment, despite affecting the amplitude (Fig. 3e and f). As the circadian clock has rhythmic robustness due to multiple feedback loops consisting of diverse transcription factors, the defect in clock regulation by *hsfb2b* seems to be minor for the *VIN3* rhythm^54^.

Under natural conditions, where environmental changes markedly occur, plants must avoid and distinguish between uncertain signals. For vernalization, plants must distinguish transient changes in temperature from winter cold. For example, plants often experience a sudden cold during late fall or a sudden warmth in early spring. Thus, plants must have an elaborate mechanism to regulate *VIN3* expression in response to ever-changing environmental conditions. Consistently, *VIN3* has been demonstrated to display dynamic expression patterns depending on fluctuating temperature^16^. For such elaborate regulation of *VIN3*, *HsfB2b* may provide a fine-tuning mechanism to prevent unintentional flowering from sudden cold.

## Materials and methods

### Plant materials and growth conditions

All *Arabidopsis thaliana* lines used were in the Columbia (Col-0) background except L*er* ecotype used to generate mapping population for map-based gene cloning. The wild-type, Col:*FRI*^*Sf2*^ (*FRI* Col) have been previously described^34^. *hsfb1, hsfb2b*, and *hsfb1 hsfb2b* mutants have been previously described^29^.

To produce *pHsfB2b::HsfB2b-eGFP* construct, the genomic sequences including 2624 bp upstream of the promoter and the whole coding sequence of *HsfB2b* were amplified by polymerase chain reaction. The fragment was cloned into *pCR2.1-TOPO* vector, then fused in-frame to *pCAMBIA1300* vector containing eGFP. The construct was transformed into the *hov1* mutant. To produce *pHsfB2b::Hsfb2b-myc*, the 3 kb *HsfB2b* promoter and the HsfB2b-coding sequence were amplified and fused in-frame to *pPZP221* vector containing 4xmyc (EQKLISEEDL). The construct was transformed into the indicated lines using Agrobacterium (*Agrobacterium tumefaciens*)-mediated *Arabidopsis* floral dip method^55^.

The plants were grown under 16 h/8 h light/dark cycle (long day) or 8 h/16 h light/dark cycle (short day) (22 °C/20 °C) in a controlled growth room with cool white fluorescent lights (125 μmol m^−2^ s^−1^). Vernalization treatments were done as previously described^36^. Nonvernalized seedlings were grown for 11 d. For 10V, 20V, 40V treatments, seedlings were grown for 10, 9, 7 d under short days respectively after germination, then transferred to vernalization chamber at 4 °C. After vernalization treatment, seedlings were sampled or transplanted to the soil. Flowering time was measured by counting the number of rosette leaves when the first flower opened using at least 20 plants.

Devernalization treatment was performed following the previously described method with some modifications^56^. Seeds were sown on a round plate containing half-strength Murashige and Skoog medium with 1% sucrose in 1% agar. The plates were wrapped with aluminum foils for complete darkness and placed at 4 °C for vernalization treatment. After 40 days of vernalization treatment, plates were transferred to heating incubator (30 °C) for additional 7 days, or placed at room temperature. Then, aluminum foils were peeled from the plates, and the plates were placed at room temperature. After 10 days of growth, seedlings were sampled or transplanted to the soil for further growth. All the plant materials and methods used in the current study were carried out following relevant institutional, national, and international guidelines and legislation.

### EMS mutagenesis and positional cloning

EMS mutagenesis was performed as previously described^57^. For the positional cloning of the causative gene of *hov1*, F2 progenies were obtained by crossing *hov1* to L*er*. Mapping procedure was followed using 135 GUS-hypersensitive F2 plants and molecular makers described as previous reports^58,59^. After rough mapping, the genomes of *hov1* and the parental − 0.2 *kb pVIN3_U_I::GUS* were sequenced and compared by illumina Hiseq2000 platform (illumina) sequencing to find mutant-specific SNPs in *hov1* using BGI services.

### Histochemical GUS staining

GUS staining was done following the standard methods that have been previously described^60^. Photographs were taken with a USB digital‐microscope Dimis‐M (Siwon Optical Technology, South Korea).

### Quantitative PCR

For real-time quantitative PCR, total RNA was isolated using TRIzol solution (Sigma). Four micrograms of total RNA were treated with recombinant DNase I (TaKaRa, 2270A) to eliminate genomic DNA. cDNA was generated using the RNA with reverse transcriptase (Thermo scientific, EP0441) and oligo(dT). Quantitative PCR was performed using the 2 × SYBR Green SuperMix (Bio-Rad 170-8882) and monitored by the CFX96 real-time PCR detection system. The relative transcript level of each gene was determined by normalization of the resulting expression levels compared to that of UBC. The primer sequences used in real-time RT-PCR analyses were shown in Supplementary Table S1.

### Immunoblotting

For immunoblot assay, the seedlings of *pHsfB2b::HsfB2b-eGFP* were harvested at each time point. Total proteins were prepared from 100 mg of harvested samples in protein extraction buffer (50 mM Tris–Cl pH 7.5, 150 mM NaCl, 10 mM MgCl_2_, 1 mM ethylenediaminetetraacetic acid (EDTA), 1% Triton X-100, 1 mM phenylmethylsulfonyl fluoride (PMSF), 1 mM 1,4-Dithiothreitol (DTT), 1Χ complete Mini, and EDTA-free protease inhibitor cocktail (Roche). Total proteins were separated by sodium-dodecyl sulfate (SDS)-PAGE. For phosphatase assay, total proteins were treated with or without alkaline phosphatase (Thermo scientific, EF0652) for 1 h at 37 °C, then separated by SDS-PAGE. The proteins were transferred to PVDF membranes (Amersham Biosciences) and probed with anti-GFP (Clontech, JL-8, 1:10,000 dilution) or anti-myc (Santa Cruz Biotechnology, sc-40, 1:10,000 dilution) antibodies overnight at 4 ℃. The samples were then probed with horseradish peroxidase-conjugated anti-mouse IgG (Cell Signaling, #7076, 1:10,000 dilution) antibodies at room temperature. The signals were detected using ImageQuant LAS 4000 (GE Healthcare) with WesternBrightTM Sirius ECL solution (Advansta).

### Confocal laser-scanning microscopic (CLSM) analysis

For microscopic observations, 5-day-old *pHsfB2b::HsfB2b-eGFP* seedlings with or without 5 days of additional cold treatment were prepared. Seedlings were pre-stained with propidium iodide (PI), mounted on glass slides, and observed using confocal microscopy (LSM700, Zeiss) following the manufacturer's instructions.

### Promoter analysis

The promoter sequences from plant species were downloaded from GBrowse at Phytozome (phytozome-next.jgi.doe.gov). The following *VIN3* loci (At5g57380) were identified using BLAST Search: *Arabidopsis lyrata* (AL8G33360), *Boechera stricta* (Bostr.26833s0518) and *Capsella rubella* (Carub.0008s1790). The sequences were processed and aligned in T-coffee (tcoffee.crg.eu).

### Yeast one-hybrid assay

Yeast one-hybrid assay was performed following the previously described method with some modifications^15^. For the reporter constructs used in the Y1H analysis, four tandem repeats containing HSE_VIN3_ (5**′**-TTAGAAACATCTAGAAAAAACAAA-3**′**) were cloned into the *pHisi* vector. For the effector, the coding sequences of *HsfA1a, HsfA2, HsfA4a, HsfA6a, HsfA8, HsfB1, HsfB2b* and *HsfC1* were cloned in-frame with the sequences of the GAL4 activation domain into *pGADT7*. The Y1H assay was performed following the manufacturer’s instructions. In brief, the reporter construct and effector construct were transformed into yeast strain YM4271. The yeast cells were spotted on synthetic define (SD) medium lacking Leu, Ura, and His, with or without 5-mM 3-amino-1,2,4-triazole (3-AT).

### Preparation of fusion protein and electrophoretic mobility shift assays (EMSA)

Coding sequence encoding DNA binding domain of *HsfB2b* was fused to *pMAL-c2* vector. The MBP and MBP-HsfB2b^DBD^ proteins were expressed in *Escherichia coli* BL21 strain according to the manufacturer’s instructions using the pMAL Protein Fusion and Purification System (#E8200; New England BioLabs) and purified using MBPtrap HP column (Cytiva) attached to ÄKTA FPLC system (Cytiva). The Cy5-labeled probes (HSE, 5′-Cy5- TTTCCTCCTTA**GAA**AC**ATC**TA**GAA**AAAACAAAAGGAGAGA-3′; mHSE, 5′-Cy5- TTTCCTCCTTA**AAA**AC**ATT**TA**AAA**AAAACAAAAGGAGAGA -3′) and unlabeled competitors were generated by annealing 40 bp-length oligonucleotides. 5 μM of purified proteins and 100 nM of Cy5-labeled probe were incubated at room temperature in binding buffer (10 mM Tris–HCl (pH 7.5), 50 mM NaCl, 1 mM EDTA, 5% glycerol and 5 mM DTT). For competition assay, 100-fold molar excess of each competitor was added to the reaction mixture before incubation. The reaction mixtures were resolved by electrophoresis through 6% polyacrylamide gel in 0.5X Tris–borate EDTA buffer at 100 V. The Cy5 signals were detected using WSE-6200H LuminoGraph II (ATTO).

### Chromatin Immunoprecipitation

Approximately 4 g of whole *Arabidopsis* seedlings were collected and cross-linked using 1% (v/v) formaldehyde for 10 min and quenched by 0.125 M glycine for 5 min under vacuum. Seedlings were rinsed with distilled water, frozen in liquid nitrogen, and grounded to fine powder. The powder was resuspended in Nuclei Isolation Buffer [1 M hexylene glycol, 20 mM PIPES-KOH (pH 7.6), 10 mM MgCl_2_, 15 mM NaCl, 1 mm EGTA, 1 mM PMSF, complete protease inhibitor mixture tablets (Roche)], and *Arabidopsis* nuclei were isolated by centrifugation, lysed by Nuclei Lysis Buffer [50 mM TRIS–HCl (pH 7.4), 150 mM NaCl, 1% Triton X-100, 1% SDS], and sonicated using a Branson sonifier to shear the DNA. Sheared chromatin solution was diluted tenfold with a ChIP Dilution Buffer [50 mM TRIS–HCl (pH 7.4), 150 mM NaCl, 1% Triton X-100, 1 mM EDTA]. The beads, chromatins and GFP-Trap A beads (gta-20, ChromoTek, Planegg, Germany) or Binding control agarose (bab-20) were mixed and incubated for overnight at 4 °C. Beads were washed with ChIP dilution buffer for 4 times and DNA extraction was performed using Chelex 100 resin following the manufacturer’s instruction. qPCR analysis was performed using 1% input and immunoprecipitated DNA.

### Accession numbers

The Arabidopsis Genome Initiative locus identifiers for the genes discussed in this paper are as follows: *VIN3* (At5g57380), *FLC* (At5g10140), *FRI* (At4g00650), *HsfA1a* (At4g17750), *HsfA2* (At2g26150), *HsfA3* (At5g03720), *HsfA4a* (At4g18880), *HsfA6a* (At5g43840), *HsfA8* (At1g67970), *HsfB1* (At4g36990), *HsfB2a* (At5g62020), *HsfB2b* (At4g11660), *HsfC1* (At3g24520), *CCA1* (At2g46830), *LHY* (At1g01060), and *PP2A* (At1g13320).

## Supplementary Information

Supplementary Information.

## Data Availability

All data generated or analyzed during this study are included in this published article and its supplementary information files.
