# Supplementary material for: HEAT SHOCK TRANSCRIPTION FACTOR B2b acts as a transcriptional repressor of VIN3, a gene induced by long-term cold for flowering
Source: Sci Rep. 2022 Jun 29;12:10963. doi: 10.1038/s41598-022-15052-6 (PMC9243095; doi:10.1038/s41598-022-15052-6)
Supplement: Supplementary file 1 — Supplementary Information. [file 41598_2022_15052_MOESM1_ESM.pdf]

Supplementary Information for

HEAT SHOCK TRANSCRIPTION FACTOR B2b Acts as a Transcriptional Repressor of *VIN3*, a Gene Induced by Long-Term Cold for Flowering

Goowon Jeong, Myeongjune Jeon, Jinwoo Shin, and Ilha Lee*

*Corresponding Author: ilhalee@snu.ac.kr

This document file includes:

Supplementary Table S1

Supplementary Figures. S1 to S8

Supplementary Table S1. Primers names and their sequences used for vector construction and quantitative PCR in this study

Oligo Name	Primer Sequence (5’-3’)	Usage
HSFB2b-GFP-F	CCATGGACCAAACAAACTTAATCAAGTG	Construct
HSFB2b-GFP-R	CCATGGTTCCGAGTTCAAGCCACGA	Construct
HSFB2b-myc-F	GTCGACACCAAACAAACTTAATCAAGTG	Construct
HSFB2b-myc-R	GTCGACTTTTCCGAGTTCAAGCCAC	Construct
4xHSE$_{VIN3}$	GCGAATTCTTAGAAACATCTAGAAAAAAACAAA<br>TTAGAAACATCTAGAAAAAAACAAATTAGAAAC<br>ATCTAGAAAAAAACAAATTAGAAACATCTAGAA<br>AAAACAAAACTAGTCG	Construct
HSFA1A-CDS-F	GCCCGGGAATGTTTGTAAATTTCAAATACTTC	Construct
HSFA1A-CDS-R	CGCTCGAGCTAGTGTTCTGTTTCTGATG	Construct
HSFA2-CDS-F	GCCCGGGAATGGAAGAACTGAAAGTGG	Construct
HSFA2-CDS-R	CGCTCGAGTTAAGGTTCCGAACCAAGAAAAC	Construct
HSFA3-CDS-F	CGCCATATGAGCCCAAAAAAAGATGCTG	Construct
HSFA3-CDS-R	CGCTCGAGCTAAGGATCATTCATTGGC	Construct
HSFA4A-CDS-F	GCCCGGGAATGGATGAGAATAATCATGGA	Construct
HSFA4A-CDS-R	CGCTCGAGTCAACTTCTCTCTGAAGAAGTC	Construct
HSFA6A-CDS-F	GCCCGGGAATGGATTATAACCTTCCAATTC	Construct
HSFA6A-CDS-R	CGCTCGAGTTATATAAAATGTTCCACTAAATC	Construct
HSFA8-CDS-F	GCCCGGGAATGGTGAAATCGACGGAC	Construct
HSFA8-CDS-R	CGCTCGAGCTATTCATTTGAAGCCAGC	Construct
HSFB1-CDS-F	GCCCGGGAATGACGGCTGTGACGGC	Construct
HSFB1-CDS-R	CGCTCGAGTTAGTTGCAGACTTTGCTGC	Construct
HSFC1-CDS-F	CGCCATATGGAGGACGACAATAGTAAC	Construct
HSFC1-CDS-R	CGCGAGCTCCTAAAAGCCACCTCGAAACAG	Construct
HSFB2B-CDS-F	GTCGACATGCCGGGGGAACAAAC	Construct
HSFB2B-CDS-R	GTCGACTCATTTTCCGAGTTCAAGCC	Construct
HsfB2b-DBD-F	CGCGAGCTCgGGAGATTCACAGAGGTCAATTC	Construct
HsfB2b-DBD-R	CGCGGATCCttaAGAGATTTTCCGCCGTTGAATA	Construct
PP2A-F	TATCGGATGACGATTCTTCGTGCAG	qRT-PCR
PP2A-R	GCTTGGTCGACTATCGGAATGAGAG	qRT-PCR
UBC-F	TTGTGCCATTGAATTGAACCC	qRT-PCR
UBC-R	CATCCTAATGTTCATTTCAAGACAG	qRT-PCR
VIN3-F	GTTTCAGGACAAGGTGACAAG	qRT-PCR
VIN3-R	TTCCCCTGAGACGAGCATTC	qRT-PCR
HSFB2b-F	CAGCTCAATACTTACGGATTTCG	qRT-PCR
HSFB2b-R	CCGTTGAATATCCCGAAGCAG	qRT-PCR
FLC-F	AGCCAAGAAGACCGAACTCA	qRT-PCR
FLC-R	TTTGTCCAGCAGGTGACATC	qRT-PCR

VIN3_A-F	TAACGGAACTTCTCATTTTCATATG	ChIP-qPCR
VIN3_A-R	TTTGCATCATCTACGTTAATTTGTG	ChIP-qPCR
VIN3_B-F	TCGAACATATAGTAGTGAGTCATA	ChIP-qPCR
VIN3_B-R	CGTTGGAAATATCTTCACGTGC	ChIP-qPCR
VIN3_C-F	GTGTTCTTCATCATCGTAAGTG	ChIP-qPCR
VIN3_C-R	GCCGAGATCCGATTTCACAC	ChIP-qPCR
VIN3_D-F	AGTACACTGGTCTTAACAAACC	ChIP-qPCR
VIN3_D-R	CGTATCATCGCATCCAAGCG	ChIP-qPCR
Cy5-HSE-F	5'Cy5-TTTCCTCCTTAGAAACATCTAGAAAAAACAAAAGGAGAGA	EMSA
HSE-R	TCTCTCCTTTTGTTTTTTCTAGATGTTTCTAAGGAGGAAA	EMSA
HSE-F	TTTCCTCCTTAGAAACATCTAGAAAAAACAAAAGGAGAGA	EMSA
Cy5-mHSE-F	5'Cy5-TTTCCTCCTTAAAAACATTTAAAAAAAACAAAAGGAGAGA	EMSA
mHSE-R	TCTCTCCTTTTGTTTTTTTTAAATGTTTTTAAGGAGGAAA	EMSA
mHSE-F	TTTCCTCCTTAAAAACATTTAAAAAAAACAAAAGGAGAGA	EMSA

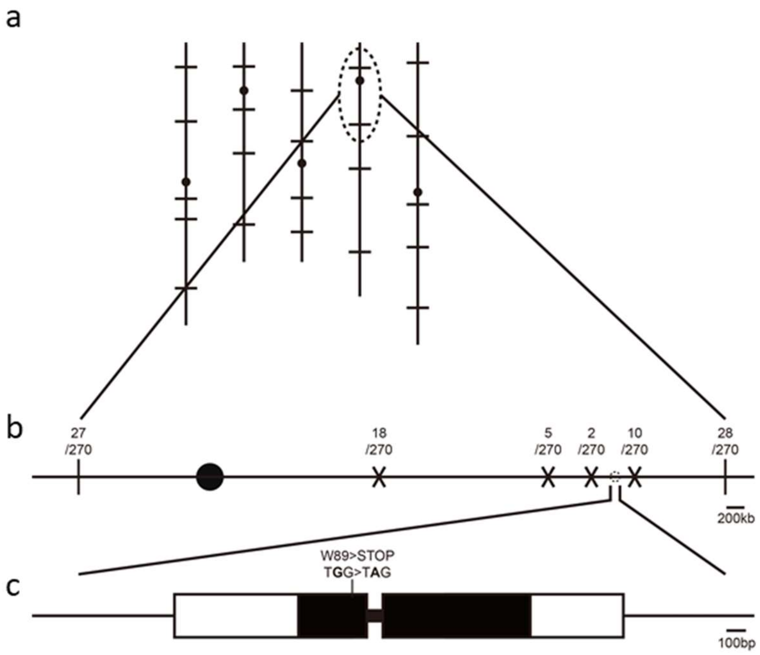

Supplementary Figure S1. Map-based cloning reveals the causative gene of the *hov1* mutation

Simplified map-based cloning procedure for *hov1*.

(a) Schematic of the *Arabidopsis* chromosomes. Vertical lines represent five *Arabidopsis* chromosomes, horizontal lines represent molecular markers, and black circles indicate centromeres. The length of each chromosome was determined based on a recombinant inbred map.

(b) Genetic intervals and molecular markers. The uppermost numbers are the number of recombinants among the 270 chromatids analysed. Vertical lines and X-signs represent molecular markers.

(c) Schematic of the *HsfB2b* structure. Black bars indicate exons and white boxes and lines represent untranslated regions and introns, respectively.

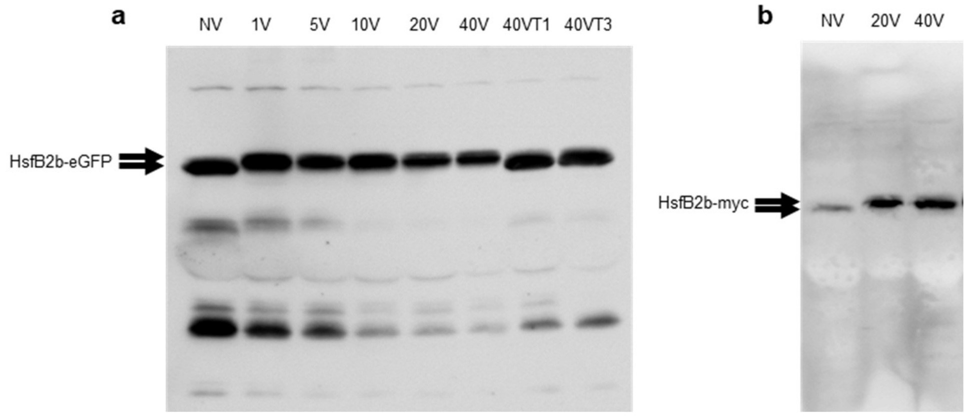

Supplementary Figure S2. Immunoblot analysis of HsfB2b-eGFP or HsfB2b-myc protein extracted from vernalized seedlings of *pHsfB2b::HsfB2b-eGFP* or *pHsfB2b::HsfB2b-myc*

(a) Biological replicate of figure 3b, showing migration of HsfB2b-eGFP upon cold exposure (b) Additional immunoblot result using pHsfB2b::HsfB2b-myc, showing migration of HsfB2b-myc upon cold exposure. Arrows denote HsfB2b-eGFP (a) and HsfB2b-myc (b) proteins.

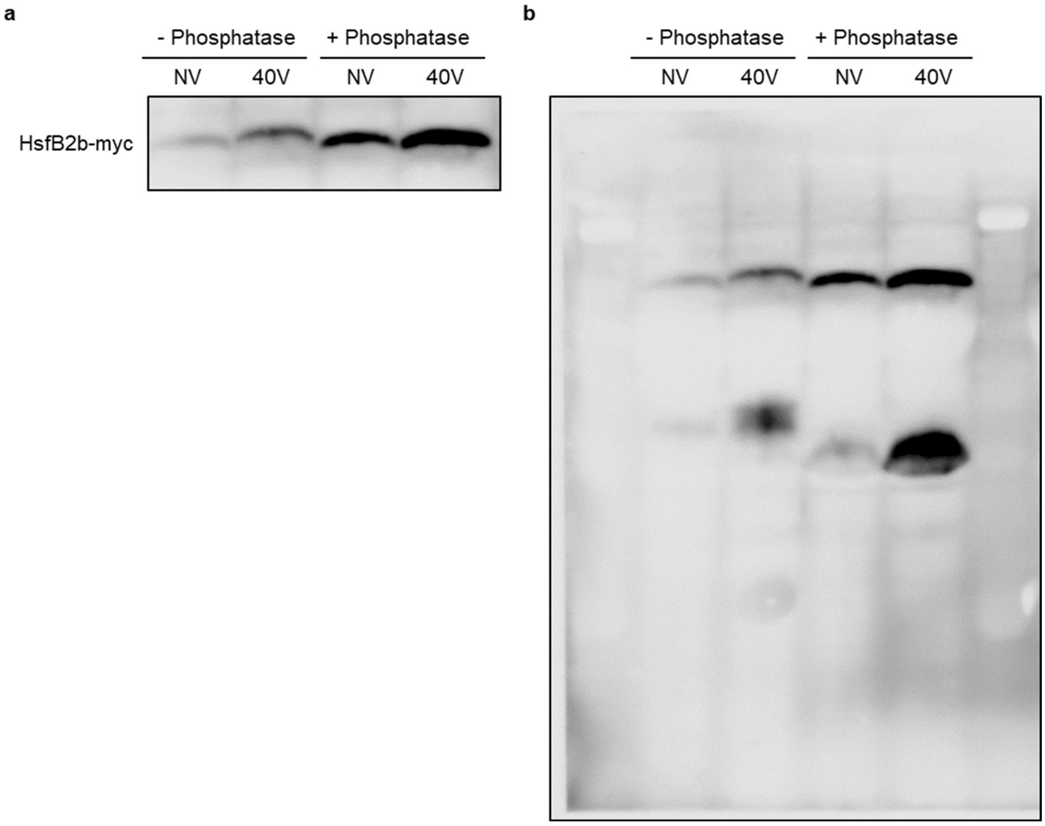

Supplementary Figure S3. HsfB2b is phosphorylated by vernalization treatment

(a) Total protein extracts were extracted from vernalized and non-vernalized seedlings of pHsfB2b::HsfB2b-myc. Extracts were treated with or without phosphatase for 1 hour at 37°C, and then separated by SDS-PAGE and immunoblot was performed using anti-myc andibody. NV, Non-vernalized; 40V, 40 d vernalized. (b) Original images used for producing (a) were displayed.

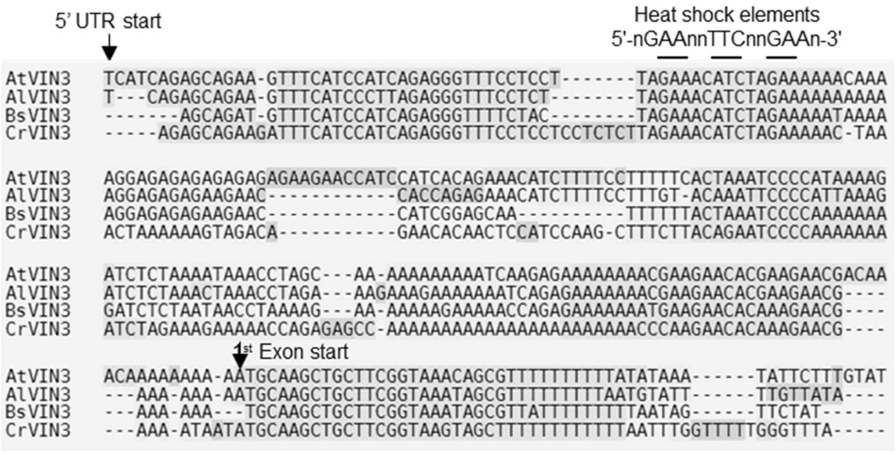

Supplementary Figure S4. HSE$_{VIN3}$ is conserved among *Arabidopsis* relatives

Multiple sequence alignment of the *VIN3* orthologs from the *Brassica* family. The region shown is +1 to +269 bp (relative to the transcription start site). At, *Arabidopsis thaliana*; Al, *Arabidopsis lyrata*; Bs, *Boechera stricta*; Cr, *Capsella rubella*. Shading represents the degree of similarity.

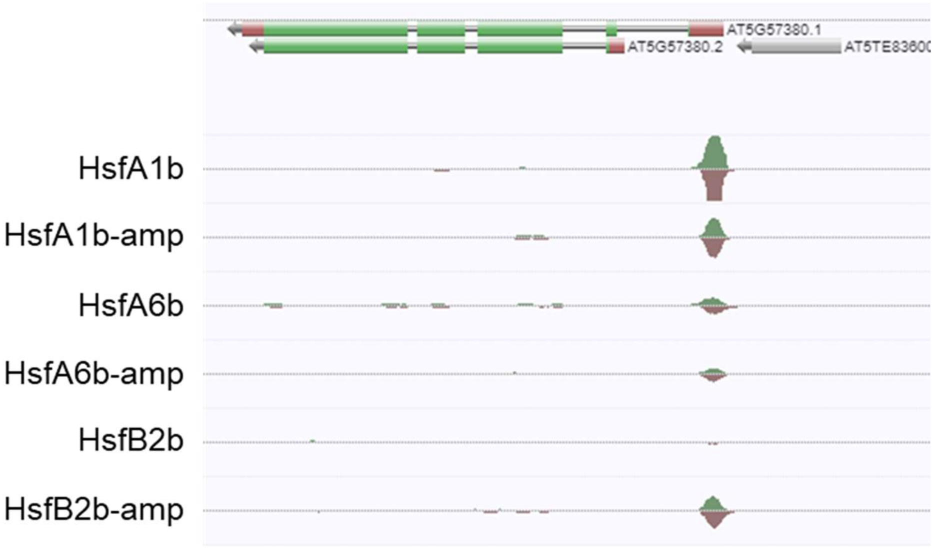

Supplementary Figure S5. DNA Affinity Purification (DAP)-seq reveals *in vitro* binding of HsfB2b to *VIN3*

DAP-seq results revealed enrichment of the Hsf proteins at the *VIN3* locus obtained from the Plant Cistrome Database (http://neomorph.salk.edu/dev/pages/shhuang/dap_web/pages/index.php (O'Malley, Huang et al. 2016)).

The top bars represent the structures of the alternatively spliced forms of the *VIN3* transcript. The green, grey, and red bars indicate exons, introns, and UTRs of *VIN3*, respectively. The peaks below represent the positions of Hsf enrichment. Hsf-amp: Amplification of DAP-seq results.

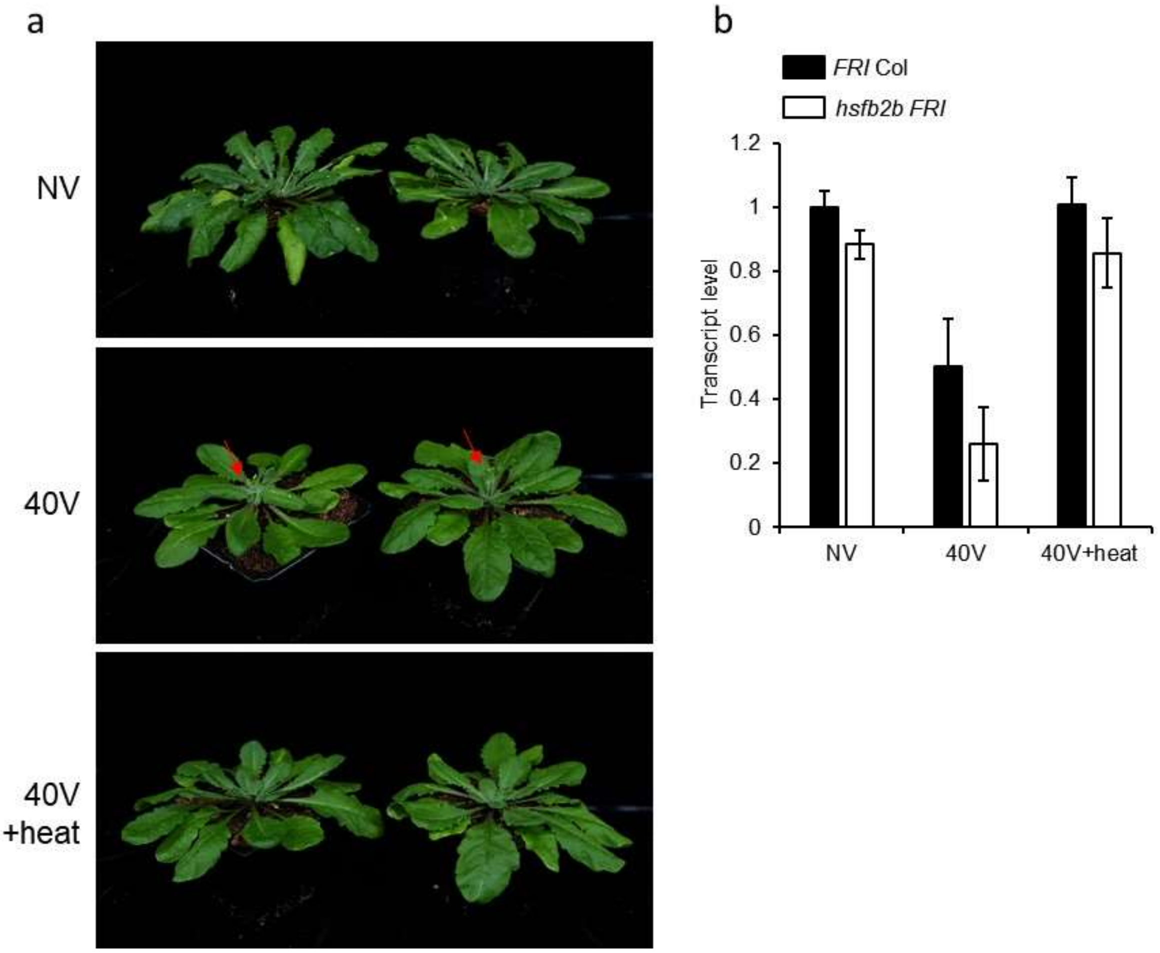

Supplementary figure S6. *hsfb2b* mutation does not change devernalization response

(a) Photographs of non-vernalized (NV), 40 d vernalized (40V), and heat-treated after 40 d of vernalization (40V+heat). Photos were taken when 40V WT and *hsfb2b* plants forms floral buds. Red arrows indicate floral buds.

(b) *VIN3* transcript levels in *FRI* Col and *hsfb2b FRI* during vernalization were determined by RT-qPCR. NV, Non-vernalized; 40V, 40 d vernalized; 40V+heat, 7 d incubated at 30°C after 40 d of cold exposure. Transcript levels were normalized to *UBC*. Data are shown as means ± SEM for three biological replicates.

a **Anti-GFP (HsfB2b-GFP)**

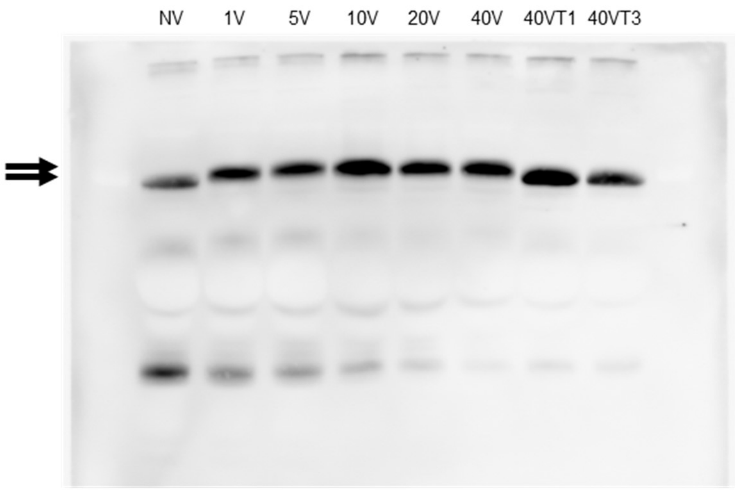

b **Ponceau S staining (Rubisco)**

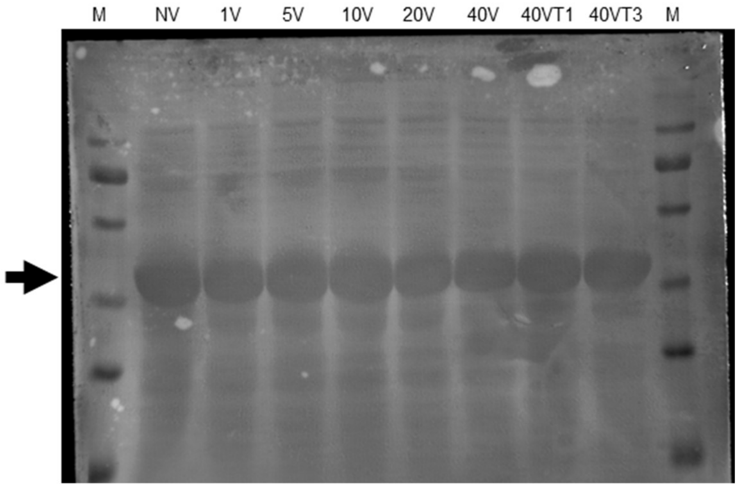

Supplementary Figure S7. Original images of representative immunoblot of HsfB2b-eGFP protein

Original images used for representative immunoblot figures in Fig. 3b were displayed. Arrows denote HsfB2b-eGFP (a) and rubisco (b) proteins.

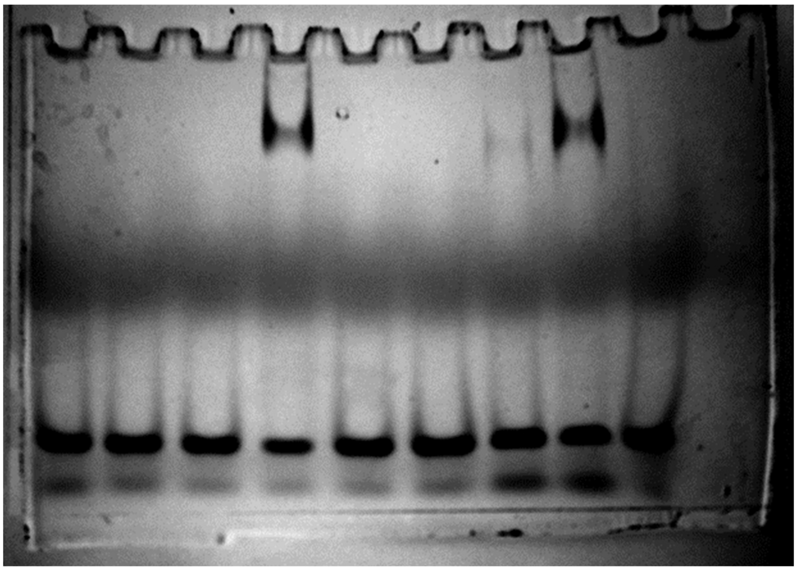

Supplementary Figure S8. Original image of representative gel

Original images used for producing representative figures in Fig. 4c were displayed.
